# Tumor Metabolism, the Ketogenic Diet and β-Hydroxybutyrate: Novel Approaches to Adjuvant Brain Tumor Therapy

**DOI:** 10.3389/fnmol.2016.00122

**Published:** 2016-11-16

**Authors:** Eric C. Woolf, Nelofer Syed, Adrienne C. Scheck

**Affiliations:** ^1^Neuro-Oncology Research, Barrow Brain Tumor Research Center, Barrow Neurological Institute, St. Joseph’s Hospital and Medical CenterPhoenix, AZ, USA; ^2^School of Life Sciences, Arizona State UniversityTempe, AZ, USA; ^3^The John Fulcher Molecular Neuro-Oncology Laboratory, Division of Brain Sciences, Imperial College LondonLondon, UK

**Keywords:** glioblastoma, glioma, cancer, metabolism, ketogenic diet, beta-hydroxybutyrate, ketones

## Abstract

Malignant brain tumors are devastating despite aggressive treatments such as surgical resection, chemotherapy and radiation therapy. The average life expectancy of patients with newly diagnosed glioblastoma is approximately ~18 months. It is clear that increased survival of brain tumor patients requires the design of new therapeutic modalities, especially those that enhance currently available treatments and/or limit tumor growth. One novel therapeutic arena is the metabolic dysregulation that results in an increased need for glucose in tumor cells. This phenomenon suggests that a reduction in tumor growth could be achieved by decreasing glucose availability, which can be accomplished through pharmacological means or through the use of a high-fat, low-carbohydrate ketogenic diet (KD). The KD, as the name implies, also provides increased blood ketones to support the energy needs of normal tissues. Preclinical work from a number of laboratories has shown that the KD does indeed reduce tumor growth *in vivo*. In addition, the KD has been shown to reduce angiogenesis, inflammation, peri-tumoral edema, migration and invasion. Furthermore, this diet can enhance the activity of radiation and chemotherapy in a mouse model of glioma, thus increasing survival. Additional studies *in vitro* have indicated that increasing ketones such as β-hydroxybutyrate (βHB) in the absence of glucose reduction can also inhibit cell growth and potentiate the effects of chemotherapy and radiation. Thus, while we are only beginning to understand the pluripotent mechanisms through which the KD affects tumor growth and response to conventional therapies, the emerging data provide strong support for the use of a KD in the treatment of malignant gliomas. This has led to a limited number of clinical trials investigating the use of a KD in patients with primary and recurrent glioma.

## Introduction

Human malignant glioma is a uniformly fatal disease due, in part, to the limitations of currently available treatments which include surgery, chemotherapy and radiation therapy. Average survival of patients with glioblastoma multiforme (GBM) is 1.5 years, and tumors of the central nervous system are the most common solid tumor in the pediatric population. It is therefore of paramount importance that new therapeutic strategies for brain cancer patients be developed, especially those that can enhance the efficacy of current treatment options without damaging normal brain tissue. Advances in our understanding of the biology of these tumors have led to an increase in the number of targeted therapies in preclinical and clinical trials (Roesler et al., 2010; Nicholas et al., 2011; Niyazi et al., 2011). While these therapies may prove somewhat effective, the heterogeneity of this tumor often precludes the targeted molecules from being found on all cells in the tumor thus reducing the efficacy of these treatments. In contrast, one trait shared by virtually all tumor cells is altered metabolism.

## Tumor Metabolism

**Figure 1:**
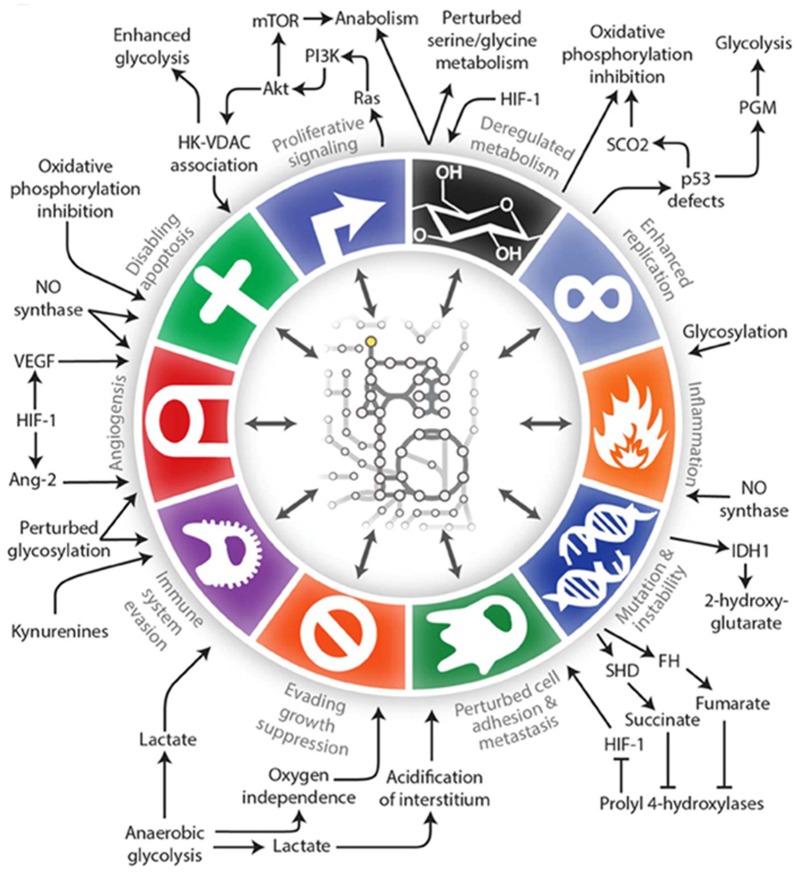
**An illustration of the interconnections between tumor metabolism with Hanahan and Weinberg’s Hallmarks of Cancer (Lewis and Abdel-Haleem, 2013)**.

Alterations in the metabolism of cancer cells, what we now call the “Warburg effect” or aerobic glycolysis, was first described by Warburg et al. (1927). Cancer cells use glycolysis to provide energy and biomolecules regardless of the availability of oxygen. This results in the production of fewer ATP molecules per molecule of glucose, and thus tumor cells require large amounts of glucose. This shift towards increased glycolytic flux in the cytosol and away from the tricarboxylic acid cycle and oxidative phosphorylation in the mitochondria occurs very early in tumorigenesis. This allows for a rapid cell proliferation even under conditions of hypoxia and in the presence of dysfunctional mitochondria. Since Warburg’s discovery, metabolism has been of interest in the cancer field but it was often overshadowed by discoveries of oncogenes, tumor suppressor genes, growth factor pathways, molecular subtypes of cancers, etc. There is a resurgence of interest in metabolism as a central theme in cancer, and we continue to find that metabolic pathways intersect and often regulate key components of tumor initiation, progression and therapy response (Clark et al., 2016; Pavlova and Thompson, 2016). In fact, altered metabolism itself has been referred to as a hallmark of cancer (Hanahan and Weinberg, 2011; Cantor and Sabatini, 2012; Ward and Thompson, 2012) in addition to being involved in virtually all of the cancer hallmarks described in the seminal article by Hanahan and Weinberg (2011) (Figure 1; Lewis and Abdel-Haleem, 2013).

The term “metabolic remodeling” has been used to describe metabolic changes that can occur in cancer cells (Obre and Rossignol, 2015), and oncogene associated pathways are now known to intersect with, and alter metabolic pathways. For example, the tumor suppressor protein p53 which plays a pivotal role in the cellular responses to hypoxia, DNA damage and oncogene activation is now known to regulate glycolysis and assist in maintaining mitochondrial integrity (Olovnikov et al., 2009; Madan et al., 2011; Puzio-Kuter, 2011; Kim and Kim, 2013; Iurlaro et al., 2014; Barron et al., 2016). Another important connection between metabolism and tumor growth is through regulation of c-MYC. Over-expression of c-MYC occurs in a wide variety of cancers including gliomas. c-MYC is a multi-functional transcription factor and the list of its target genes include those involved in both cell proliferation and cell metabolism (Miller et al., 2012; Zwaans and Lombard, 2014; Altman et al., 2015; Hsieh et al., 2015; Stine et al., 2015). In addition to stimulating glycolysis, c-MYC has been found to activate glutaminolysis and lipid synthesis from citrate (Obre and Rossignol, 2015).

With the advent of molecular analyses, studies of growth factor pathways seemed to overshadow the influence of metabolism on cancer growth. Over-activation of the stress responsive PI3K/AKT signaling pathway is typical in many cancers and often due to activation of growth factor signaling pathways involved in glioma growth such as platelet-derived growth factor, epidermal growth factor and insulin growth factor. We now know that these growth factor pathways are intertwined with metabolic signally pathways (Iurlaro et al., 2014; Martini et al., 2014; Courtnay et al., 2015; Dibble and Cantley, 2015; Roberts and Miyamoto, 2015). PI3K/AKT signaling has been closely linked to metabolism and under low glucose conditions results in rapid tumor cell death (Robey and Hay, 2009; Yang et al., 2009; Marie and Shinjo, 2011).

Another important “hub” linking metabolism and cancer is hypoxia-inducible factor 1 (HIF-1). HIF-1 expression is activated by hypoxia, which is typically found in high grade gliomas and other cancers. HIF-1 is a heterodimeric transcription factor that induces the transcription of a variety of genes involved in angiogenesis (vascular endothelial growth factor (VEGF) and other cytokines) in an attempt to improve tissue perfusion. This results in the formation of abnormal blood vessels that can increase inflammation and edema in brain tumors, as well as induction of the transcription of a variety of genes that promote invasion, migration and tumor growth (Fischer et al., 2002; Kaur et al., 2005; Fujiwara et al., 2007; Hayashi et al., 2007; Mou et al., 2010; Horing et al., 2012; Proescholdt et al., 2012; Yang et al., 2012; Masson and Ratcliffe, 2014; Justus et al., 2015). In addition to specific actions that relate to the tumor cell’s response to oxygen availability, HIF-1 interacts with the PI3K/AKT signaling path to act as a regulator of cancer metabolism, proliferation and glycolysis (Pore et al., 2006; Wei et al., 2013; Courtnay et al., 2015; Justus et al., 2015). It also affects the activation of nuclear factor-kappa B (NF-κB), a transcriptional activator that is central to the regulation of various signal transduction pathways and to transcriptional activation events that mediate inflammation, cell proliferation, cell migration and angiogenesis. HIF-1 may, at least in part, provide the molecular basis for the Warburg effect by “reprograming” cellular metabolism in response to oxygen availability (Corbet and Feron, 2015; Courtnay et al., 2015). HIF-1 also is a central figure in alterations to the tumor microenvironment which not only affects tumor cell growth, but also response to therapy (Yamada et al., 1999; Joon et al., 2004; Amberger-Murphy, 2009; Dewhirst, 2009; Hattingen et al., 2011; Metallo et al., 2011; Yang et al., 2012; Danhier et al., 2013; Justus et al., 2015).

It is clear that cancer cell metabolism is far more complex than originally thought. A number of cancer associated mutations affect metabolism and defects in mitochondria are seen in cancer that also link metabolism with cancer initiation and progression. Recent studies have shown that changes in cellular metabolism can alter the expression of specific microRNAs and promote epigenetic changes in tumor cells (Arora et al., 2015; Bishop and Ferguson, 2015; Chan et al., 2015). Although some of these interactions are mentioned above, in-depth discussions of all of the interactions that occur between cancer and metabolism are beyond the scope of this review and the reader is referred to a number of reviews on these subjects (Gatenby and Gillies, 2004; Vander Heiden et al., 2009; Cantor and Sabatini, 2012; Ward and Thompson, 2012; Semenza, 2013; Gaude and Frezza, 2014; Masson and Ratcliffe, 2014; Boroughs and DeBerardinis, 2015; Casey et al., 2015; Robey et al., 2015; Asati et al., 2016; Barron et al., 2016; Bost et al., 2016; Molon et al., 2016; Pavlova and Thompson, 2016; Pérez-Escuredo et al., 2016). The fact that metabolic dysregulation is seen in virtually all tumor cells has led to suggestions that a promising therapeutic strategy may be to exploit this feature. One potential way to achieve this goal is through the use of the therapeutic ketogenic diet (KD) or physiologically similar methods, such as caloric restriction (CR) or intermittent fasting.

## The Ketogenic Diet: Overview and Preclinical Evidence

The KD is a high-fat low protein/carbohydrate diet used to treat refractory epilepsy (Kim and Rho, 2008; Cross, 2013). It has been shown to have neuroprotective effects and there are now studies to determine its efficacy for a number of neurological disorders, including epilepsy, Alzheimer’s disease, Parkinson’s disease, sleep disorders, headache, traumatic brain injury, amyotrophic lateral sclerosis, pain and autism (Masino and Ruskin, 2013; Gano et al., 2014). The KD increases blood ketones and decreases blood glucose by simulating the physiological response to fasting, thus leading to high rates of fatty acid oxidation and an increase in the production of acetyl coenzyme A (acetyl-CoA). When the amount of acetyl-CoA exceeds the capacity of the tricarboxylic acid cycle to utilize it, there is an increase in the production of the ketone bodies β-hydroxybutyrate (βHB) and acetoacetate (ACA), which can be used as an energy source in the normal brain (Veech et al., 2001; Cahill and Veech, 2003; Vanitallie and Nufert, 2003; Morris, 2005; Gasior et al., 2006). Since normal cells readily use ketones as an alternate energy source, they are unlikely to be adversely affected by reduced glucose. In contrast, the metabolic alterations found in cancer cells are generally thought to reduce their ability to be “flexible” regarding their primary energy source, and thus they require glucose (Tisdale and Brennan, 1983; Seyfried and Mukherjee, 2005; Zhou et al., 2007; Maurer et al., 2011; Seyfried et al., 2011; Seyfried, 2012). By reducing the glucose availability to cancer cells and providing ketones as an alternative energy source for normal cells, the KD may target the Warburg Effect in highly glycolytic tumors, such as malignant gliomas.

The use of metabolic alteration for the therapy of brain tumors has been championed by Seyfried et al. (2011). They used the VM (Shelton et al., 2010) and CT-2A (Marsh et al., 2008) mouse tumor models to show that a KD, especially when given in restricted amounts, extends survival. D’Agostino and co-workers have added hyperbaric oxygen and ketone supplementation to demonstrate reduced tumor cell growth and metastatic spread in the VM metastatic tumor model (Poff et al., 2014, 2015). We used the syngeneic intracranial GL261-luc/albino C57/Bl6 model to demonstrate that CR was not necessary for the anti-tumor effects of the KD (Stafford et al., 2010), particularly when a 4:1 fat:carbohydrate plus protein formulation is used (Scheck et al., 2012; Woolf et al., 2015; Lussier et al., 2016). Recently Martuscello et al. (2016) demonstrated inhibition of glioma stem cell growth *in vitro* and *in vivo* through the use of a supplemented high fat low carbohydrate diet.

The KD and similar diets used as a monotherapy have a pluripotent effect on the growth on tumors both *in vitro* and *in vivo* which may depend, at least in part, on the model system, the specific metabolic intervention and the molecular underpinnings of the tumor itself (Freedland et al., 2008; Otto et al., 2008; Mavropoulos et al., 2009; Stafford et al., 2010; Kim H. S. et al., 2012; Caso et al., 2013; Poff et al., 2013, 2015; Simone et al., 2013; Lv et al., 2014; Shukla et al., 2014; Hao et al., 2015; Woolf et al., 2015). In addition, the exact composition of the diet may also alter its effects, and there are studies in some cancers looking specifically at polyunsaturated fatty acids (PUFAs), particularly the omega-3 class, for their anti-cancer properties (Sauer et al., 2007; Pifferi et al., 2008; Wang et al., 2012, 2016; Hofmanova et al., 2013; Abel et al., 2014). The striking feature of the work done to date in a number of model systems using different dietary interventions is that alterations in metabolism have a far reaching effect on tumor cells, tumors and the tumor microenvironment. Studies have shown reductions in growth rate as one might expect however, there are also changes in the formation of reactive oxygen species and oxidative stress (Stafford et al., 2010; Milder and Patel, 2012; Allen et al., 2013), angiogeneisis (Zhou et al., 2007; Jiang and Wang, 2013; Woolf et al., 2015), hypoxia (Maurer et al., 2011; Poff et al., 2015; Woolf et al., 2015), inflammation and peri-tumoral edema (Mavropoulos et al., 2009; Woolf et al., 2015), metastasis and invasion (Gluschnaider et al., 2014; Lv et al., 2014; Hao et al., 2015; Poff et al., 2015) and the expression of various transcriptional and post-transcriptional modulators such as NF-κB (Woolf et al., 2015) and microRNAs (Pazmandi et al., 2015).

## KD in Combination with Standard Therapies

**Figure 2:**
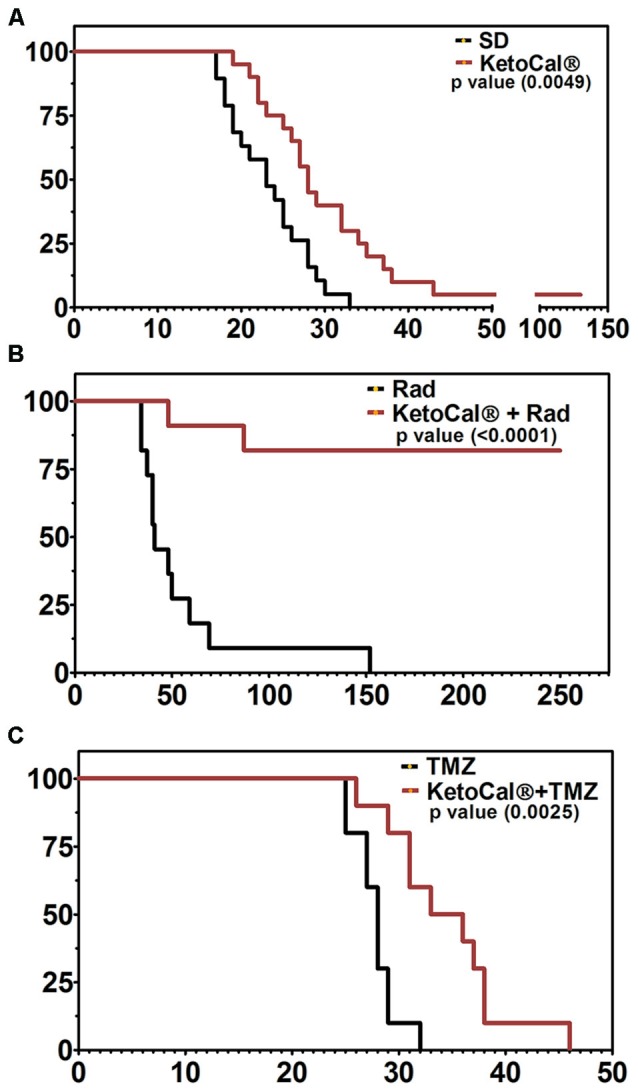
**Kaplan-Meier survival plot of animals implanted intracranially with GL261-luc2 malignant glioma cells and (A)** maintained on KetoCal^**®**^ [KC, the 4:1 fat:carbohydrate plus protein formulation of the ketogenic diet (KD)] vs. standard diet (SD); **(B)** treated with 2x4Gy radiation vs. KC plus radiation, and **(C)** treated with 50 mg/kg temozolomide (TMZ) vs. KC plus TMZ. Animals on KC survived significantly longer when treated with KC alone, when KC was combined with radiation, and when KC was combined with TMZ (Scheck et al., 2011; Abdelwahab et al., 2012).

Although evidence suggests that the KD provides anti-tumor benefits on its own, perhaps the most effective use of the KD is in combination with standard cancer therapies such as radiation and chemotherapy (Allen et al., 2014). The KD greatly enhanced survival in a mouse model of malignant glioma when combined with temozolomide (TMZ) when compared to either treatment alone (Figure 2; Scheck et al., 2011). Using a bioluminescent, syngeneic intracranial model of malignant glioma, the KD was shown to significantly potentiate the anti-tumor effect of radiotherapy. In fact, 9 out of 11 animals maintained on the KD and treated with radiation had complete and sustained remission of their implanted tumors, even after being switched back to a standard rodent diet (Figure 2; Abdelwahab et al., 2012). Allen et al. (2013) reported similar results when the KD is combined with radiation and chemotherapy in a lung cancer xenograft model. That is, they found decreased tumor growth rate and increased survival. CR and short-term fasting have also been found to be synergistic with radiation and other anti-cancer therapeutics in both preclinical and clinical studies (Raffaghello et al., 2008, 2010; Lee et al., 2010, 2012; Safdie et al., 2012; Champ et al., 2013, 2014; Saleh et al., 2013; Klement and Champ, 2014).

The effectiveness of radiation therapy is due to a number of factors including relative damage done to tumor cells vs. normal tissue and the ability of normal cells and tumor cells to repair the damage (Klement and Champ, 2014; Santivasi and Xia, 2014). KD or CR may modulate the ability of tumor and normal cells to repair radiation-induced damage (Klement and Champ, 2014). Studies have shown that CR can enhance DNA repair in normal cells (Heydari et al., 2007); however, this may not be the case in tumor cells, and the differential response of tumor cells and normal cells to genotoxic stress may be mediated by reduced Insulin-like growth factor 1 (IGF1) and glucose in the tumor cells. We and others have shown that insulin growth factor is reduced in animals maintained on a KD (Freedland et al., 2008; Mavropoulos et al., 2009; Scheck et al., 2012; Klement and Champ, 2014).

Finally, ketones and the KD have been shown to affect the immune system (Kim D. Y. et al., 2012; Husain et al., 2013; Rahman et al., 2014; Youm et al., 2015), and we have shown that the KD also reverses tumor-mediated immune suppression in a mouse model of malignant glioma (Lussier et al., 2016). As radiation-induced tumor killing is known to expose the immune system to a greater diversity of tumor antigens, it is possible that the KD as an adjuvant works to augment the effect of radiation in part by enhancing immunity against GBM.

The variety of effects seen when glucose in lowered and/or ketones are increased suggests that this may also potentiate other therapies, including newer immune- and targeted therapies. Concerns that potentiation of the anti-tumor effect of a particular therapy may also increase its effect on normal brain are valid; however, we and others have shown that the gene expression changes seen in tumor are different than those seen in normal brain (Stafford et al., 2010; Maurer et al., 2011). Further, the KD is known to have neuroprotective effects (Puchowicz et al., 2008; Lund et al., 2009; Maalouf et al., 2009; Hartman, 2012) and thus it has been postulated that this may actually help to protect the normal brain from the deleterious effects of radio and chemotherapy. Taken together, the preclinical data provides strong support for the clinical use of the KD or CR as an adjuvant therapy for the treatment of gliomas and other cancers.

## β-Hydroxybutyrate as an Anti-Cancer Agent

The ketone body βHB has traditionally been thought of as simply a metabolic substrate that replaces glucose during the KD, fasting or exercise; however, the effects of increased ketones go beyond simple considerations of energy availability (Newman and Verdin, 2014; Jaworski et al., 2016). *In vitro* investigations demonstrated that βHB is able to recapitulate, in part, the *in vivo* effects of the full KD (Skinner et al., 2009; Rossi et al., 2015). This suggests that the ketone bodies themselves possess antitumor effects, and that perhaps the effects of the KD are mediated, at least in part, by the ketone bodies. Additional evidence for this comes from data showing that the use of ketone supplementation can enhance the effects of the KD and may even be effective in some diseases when used alone (Veech, 2004, 2014; Kashiwaya et al., 2013; Poff et al., 2014; Shukla et al., 2014; Newport et al., 2015; Youm et al., 2015). Though the mechanisms are still under investigation, it is known that βHB is an endogenous Class I and IIa histone deacetylase (HDAC) inhibitor (Shimazu et al., 2013). HDACs primarily functions by deacetylating lysine residues on both histone and non-histone proteins, resulting in increased global acetylation and regulation of gene expression. In this way, βHB has the capacity to modulate the epigenetic environment within cells, which may contribute to the beneficial effect of the KD and CR.

Our own investigations into the interactions between βHB and glioblastoma cells have revealed insights into the molecular basis for some of the KD’s effects, most notably its radio- and chemo-sensitizing effects. *In vitro* studies using βHB demonstrated that, even in the presence of high glucose, physiologically relevant doses of βHB reduced proliferation of several human glioblastoma cell lines, two human cancer stem cell lines, and a murine glioma cell line. Additionally, similar treatment with βHB resulted in potentiation of low doses of ionizing radiation therapy in both sensitive and resistant populations (Rossi et al., 2015; Silva-Nichols et al., 2015). Further, in a separate study βHB potentiated the chemotherapeutic agent 1,3-bis(2-chloroethyl)-1 nitrosourea (BCNU, carmustine) in a cell line derived from a recurrent human glioblastoma (Scheck et al., 2012). Taken together, these results suggest that ketone supplementation may provide an effective, less stringent alternative to the rigors of the KD; yet additional studies are needed to further develop this approach.

## KD in Humans

Studies of glucose utilization in cancer go back prior to the 1980s, including studies of metabolism and cancer cachexia (Tisdale et al., 1987; Fearon et al., 1988). These and other studies suggested that the KD consisting of a high percentage of medium chain triglycerides (MCT) along with various supplements resulted in weight gain and improved nitrogen balance in both animals and humans. Nebeling et al. (1995) published a case report in which they used a similar KD based on MCT oil to treat two female pediatric patients with advanced stage malignant brain tumors (Nebeling and Lerner, 1995). They demonstrated that dietary induced ketosis decreased the availability of glucose to the tumor without causing a decrease in patient weight or overall nutritional status. Furthermore, both children had long-term tumor management (Nebeling et al., 1995).

The 2nd case report was published by Zuccoli et al. (2010). This patient was a 65-year-old female with a multicentric glioblastoma. She was put on a 4:1 (ratio of fats:carbohydrate plus protein) calorie restricted (600 kcal/day) KD during radiation and chemotherapy. During this time her body weight dropped by 20%, she had reduced blood glucose, increased urinary ketones and, most importantly, no observable brain tumor by either fluorodeoxyglucose Positron Emission Tomography (FDG-PET) or magnetic resonance imaging (MRI). The tumor recurred 10 weeks after the patient resumed her normal eating habits and she succumbed to her disease less than 2 years after diagnosis. While this patient did not experience long-term tumor control after cessation of the diet, this report demonstrated that the diet could be tolerated, even when used in a calorie-restricted setting. Results of a phase 1 clinical trial were reported in 2011 by a German group (Schmidt et al., 2011). Tolerability of a restricted calorie KD was tested in 16 patients with a variety of advanced (end-stage) cancers. There were no severe side effects and 5 of the 16 patients were able to complete the 3 months treatment. These five patients had stable disease while on the diet. Two of the 11 remaining patients died early following the beginning of the trial, one was unable to tolerate the diet and dropped out immediately, two patients dropped out for personal reasons, one was unable to continue the diet for more than a month and three had disease progression within less than 2 months of starting the diet and one dropped out to resume chemotherapy. While this trial demonstrated tolerability and favorable side effect profile, the antitumor efficacy could not be assessed due to the variety and severity of disease in the patients. Recently, Schwartz et al. (2015) reported on two patients with recurrent GBM treated with a calorie restricted KD as a monotherapy and although the diet was tolerated, both patients showed tumor progression—The first within 4 weeks and the second within 12 weeks of beginning the protocol. This group also hypothesized that an analysis of ketolytic and glycolytic enzyme levels in tumor tissue may help identify patients that are more likely to respond to a KD, although this has not yet been proven. More recently, a number of prospective clinical trials have been initiated which have been summarized in Table 1. These trials include studies of up-front treatment using the KD in addition to standard radiation and chemotherapy in patients diagnosed with GBM.

**Table 1:** **Active clinical trials: ketogenic diet and gliomas**.

ClinicalTrials.gov Identifier	Dates	Title	Location	Data (Enrollment)
01716468	First received: 9/18/12 Last updated: 4/9/16 Last verified: April 2016	Ketogenic Diet in Advanced Cancer PI: Jocelyn Tan, MD	VA Pittsburgh Healthcare System	Safety; long term tolerability; quality of life; tumor growth/spread; overall and progression free survival (17 patients)
02046187	First received: 1/17/14 Last updated: 3/5/15 Last verified: March 2015	Ketogenic Diet With Radiation and Chemotherapy for Newly Diagnosed Glioblastoma PI: Adrienne C Scheck, PhD Christopher Dardis, MD	St. Joseph’s Hospital and Medical Center, Phoenix	Tolerability; overall survival; time to progression; patient quality of life (QOL); caregiver quality of life; cognitive changes; seizure activity (40 patients)
01754350	First received: 12/14/12 Last updated: 3/31/15 Last verified: March 2015	Calorie-restricted, Ketogenic Diet and Transient Fasting During Reirradiation for Patients With Recurrent Glioblastoma (ERGO2) PI: Johannes Rieger, PD Dr. med.	Johann Wolfgang Goethe University Hospitals TAVARLIN (Darmstadt, Germany)	Tolerability; progression free survival (6 months after re-irradiation); overall survival; seizure frequency; QOL; depression; attention (50 patients)
02286167	First received: 11/5/14 Last updated: 11/17/14 Last verified: October 2014	Glioma Modified Atkins-based Diet in Patients With Glioblastoma PI: Jaishri O. Blakeley, MD	Sidney Kimmel Comprehensive Cancer Center, Johns Hopkins	Feasibility of MAD; cerebral glutamate and glutamine concentrations (MRS); dietary compliance (25 patients)
01535911	First received: 2/3/12 Last updated: 9/8/15 Last verified: September 2015	Pilot Study of a Metabolic Nutritional Therapy for the Management of Primary Brain Tumors (Ketones)	Michigan State University	Safety/Efficacy Study CT-PET scan will be used to measure changes in brain tumor size. Energy restricted ketogenic diet (ERKD) newly diagnosed GBM subjects.
01865162	First received: 5/24/13 Last updated: 11/25/14 Last verified: May 2013	Ketogenic Diet as Adjunctive Treatment in Refractory/End- stage Glioblastoma Multiforme: a Pilot Study PI: Pavel Klein, M.D.	Mid-Atlantic Epilepsy and Sleep Center, LLC Collaborator University of Pittsburgh	Safety; compliance (tolerability); survival; time to edema requiring steroids (6 patients)
02302235	First received: 11/24/14 Last updated: 11/25/14 Last verified: November 2014	Ketogenic Diet Treatment Adjunctive to Radiation and Chemotherapy in Glioblastoma Multiforme: a Pilot Study (GBMXRT) PI: Pavel Klein, M.D.	Mid-Atlantic Epilepsy and Sleep Center, LLC Collaborator Neuroscience Research Foundation	Survival; time to recurrence; time to radiological progression; tolerability (42 patients)

The case reports described above along with numerous anecdotal reports suggest that the KD may be a promising anti-cancer therapy; however, more work is needed to determine how to best utilize this, and other metabolic therapies for the treatment of tumors. Most of the information regarding the best way to use the KD comes from the epilepsy literature. Further research is needed to determine optimum blood ketone and glucose levels for anticancer effects. In addition, a variety of KDs are used for seizure control and it is not clear if one or more of the different formulations will provide the best results for cancer patients. Finally, while the KD has a long record of safety in the epilepsy community, side effects that occur when used in combination with cancer therapies may differ in type or severity. This data will come from carefully controlled clinical trials that include input from registered dietitians well-versed in the use of the KD. Patient enrollment into clinical trials requires “buy-in” from the medical community. Physicians must be educated on the therapeutic benefits of metabolic alteration as an adjuvant therapy. As with any decision regarding therapy, the patient’s overall condition, including nutritional status, must be taken into account. As suggested by Klement and Champ (2014), cancer patients should be assessed for nutritional needs and tolerability of such interventions.

Concern about patients’ quality of life is sometimes given as a reason not to employ KD. Compliance can be made more difficult by the use of steroids (prescribed for peritumoral edema) that often increase hunger and raise blood glucose levels. To address this, at least one clinical trial (NCT02046187) includes an analysis of both patient and caregiver quality of life. Quality of life measurements are being added to more clinical trials, as the importance of this has become recognized at the national level (van den Bent et al., 2011; Boele et al., 2013; Dirven et al., 2014). While some clinicians are concerned compliance will reduce quality of life, the patient’s that do remain on the KD often comment that this allows them to participate in their own therapy. Despite these caveats, the existing preclinical data suggesting anti-tumor efficacy and a synergistic effect with standard therapies provides a strong impetus to conduct controlled clinical trials, particularly those that will shed light on the interactions between the KD and other therapies.

## Conclusion

Improvements in the survival and quality of life for patients with malignant brain tumors require the implementation of new therapeutic modalities, especially those that increase the efficacy of current therapies without increasing toxic side effects. While the rapid accumulation of data defining the molecular and genetic aberrations present in these tumors has suggested a host of targets for the development of new therapies, targeted therapies tried to date have met with limited success. This is at least in part due to the molecular heterogeneity of these tumors that prevents any one target from being present on all cells. In contrast, metabolic dysregulation is present in virtually all tumor cells and there is increased interest in using metabolic therapies such as the KD and ketone supplementation for the treatment of various cancers, especially brain tumors. Preclinical data has demonstrated that the anti-tumor effects of the KD and CR are multi-faceted, and alterations in energy metabolism can inhibit cancer cell growth and increase the tumor’s response to therapy. This provides a strong impetus to continue work designed to elucidate the mechanisms through which the KD exerts its anticancer effects, as well as suggesting the need for the design of controlled clinical trials that will shed light on the most effective way to implement metabolic therapies in combination with standard therapies for the treatment of malignant disease. This is a novel therapeutic paradigm, and we have only begun to scratch the surface of its potential.

## Author Contributions

ECW, NS and ACS contributed to the writing of this review article.

## Funding

This work was funded in part by grants to ACS from Students Supporting Brain Tumor Research and support for ECW from the School of Life Sciences, Arizona State University. Brain Tumour Research Campaign helps to fund the work done in Dr. Nelofer Syed’s laboratory in the United Kingdom.

## Conflict of Interest Statement

The authors declare that the research was conducted in the absence of any commercial or financial relationships that could be construed as a potential conflict of interest.
